# Global burden, distribution, and interventions for infectious diseases of poverty

**DOI:** 10.1186/2049-9957-3-21

**Published:** 2014-07-31

**Authors:** Zulfiqar A Bhutta, Johannes Sommerfeld, Zohra S Lassi, Rehana A Salam, Jai K Das

**Affiliations:** 1 Center of Excellence in Women & Child Health, The Aga Khan University, Karachi, Pakistan; 2 Center for Global Child Health Hospital for Sick Children, Toronto, Canada; 3 UNDP/World Bank/WHO Special Programme for Research and Training in Tropical Diseases (TDR), Geneva, Switzerland; 4 Division of Women and Child Health, The Aga Khan University, Karachi, Pakistan

**Keywords:** Infectious diseases of poverty, Neglected tropical diseases malaria, HIV/AIDS, Tuberculosis, Community-based interventions, Community platforms, Community-health workers

## Abstract

Infectious diseases of poverty (IDoP) disproportionately affect the poorest population in the world and contribute to a cycle of poverty as a result of decreased productivity ensuing from long-term illness, disability, and social stigma. In 2010, the global deaths from HIV/AIDS have increased to 1.5 million and malaria mortality rose to 1.17 million. Mortality from neglected tropical diseases rose to 152,000, while tuberculosis killed 1.2 million people that same year. Substantial regional variations exist in the distribution of these diseases as they are primarily concentrated in rural areas of Sub-Saharan Africa, Asia, and Latin America, with geographic overlap and high levels of co-infection. Evidence-based interventions exist to prevent and control these diseases, however, the coverage still remains low with an emerging challenge of antimicrobial resistance. Therefore, community-based delivery platforms are increasingly being advocated to ensure sustainability and combat co-infections.

Because of the high morbidity and mortality burden of these diseases, especially in resource-poor settings, it is imperative to conduct a systematic review to identify strategies to prevent and control these diseases. Therefore, we attempted to evaluate the effectiveness of one of these strategies, that is community-based delivery for the prevention and treatment of IDoP. In this paper, we describe the burden, epidemiology, and potential interventions for IDoP. In subsequent papers of this series, we describe the analytical framework and the methodology used to guide the systematic reviews, and report the findings and interpretations of our analyses of the impact of community-based strategies on individual IDoPs.

## Multilingual abstracts

Please see Additional file 1 for translations of the abstract into the six official working languages of the United Nations.

## Introduction

*The Global Burden of Disease Study 2010* reports an increase of 111,000 deaths globally attributable to malaria and neglected tropical diseases (NTDs) (including chagas, leishmaniasis, African trypanosomiasis, schistosomiasis, cysticercosis, echinococcosis, dengue, rabies, ascariasis, as well as other NTDs) in the last two decades, with substantial regional variations and Sub-Saharan Africa accountable for most of the premature mortalities [1,2]. In 2010, the global deaths from human immunodeficiency virus (HIV)/ acquired immunodeficiency syndrome (AIDS) have increased to 1.5 million, and malaria mortality rose to 1.17 million. Mortality from NTDs rose to 152,000, while tuberculosis (TB) killed 1.2 million people that same year [2]. The United Nations (UN) Millennium Declaration which translated into the Millennium Development Goals (MDGs) underscored the need to combat HIV/AIDS, malaria, and TB, while other infectious diseases including most of the tropical diseases collectively termed as NTDs have slipped into a ‘neglected’ category, by default [3-6]. NTDs along with HIV, TB, and malaria are collectively referred to as ‘infectious diseases of poverty’ (IDoPs), and these are primarily concentrated in rural areas of Sub-Saharan Africa, Asia, and Latin America, with geographic overlap resulting in high levels of co-infection [7-11]. It is estimated that more than 90% of the total impact as a result of death and disability caused by neglected diseases occurs in Sub-Saharan Africa [12].

IDoP disproportionately affect the poorest populations in the world and contribute to a cycle of poverty as a result of decreased productivity ensuing from long-term illness, disability, and social stigma [3,5]. The affected populations usually have fewer material, physical, and financial resources to draw from and limited or no access to integrated health care, prevention tools and medications, thus resulting in the most severe adverse impacts. Various social determinants also compound the issue and these include gender dilemmas, unemployment, illiteracy, poor nutrition, indoor air pollution, political instability, and lack of access to proper sanitation and health education, among others [3]. The socioeconomic and physical conditions of those living in poverty create environments that facilitate the transmission of vectors and pathogens consequently leading to long-term illness that further exacerbates poverty by diminishing productivity. Furthermore, global attention and resources have been focused on HIV/AIDS, malaria, and TB as these are specifically outlined in MDG 6, while NTDs have consequently been relegated into the group of “other diseases”, and until 2010, NTDs represented only 0.6% of the total international development assistance for health [13] despite affecting as many poor people as the big three diseases.

A large proportion of these infectious diseases in low- middle- income countries (LMICs) are entirely avoidable or treatable with existing medicines or interventions [14]. Effective and simple interventions to prevent and treat these infectious diseases exist but their delivery to affected populations has proven very difficult due to weak health system infrastructure in many developing countries, thus the need to shift the focus from institutional care delivery to community platforms for improved accessibility. This paper aims to review the disease burden, distribution, existing interventions, and coverage for the prevention and control of IDoP (including NTDs, malaria, TB, and HIV/AIDS), and is followed by a series of papers evaluating the effectiveness of community delivered interventions for the prevention and control of each IDoP.

### Review: disease distribution, burden, and consequences

**Figure 1:**
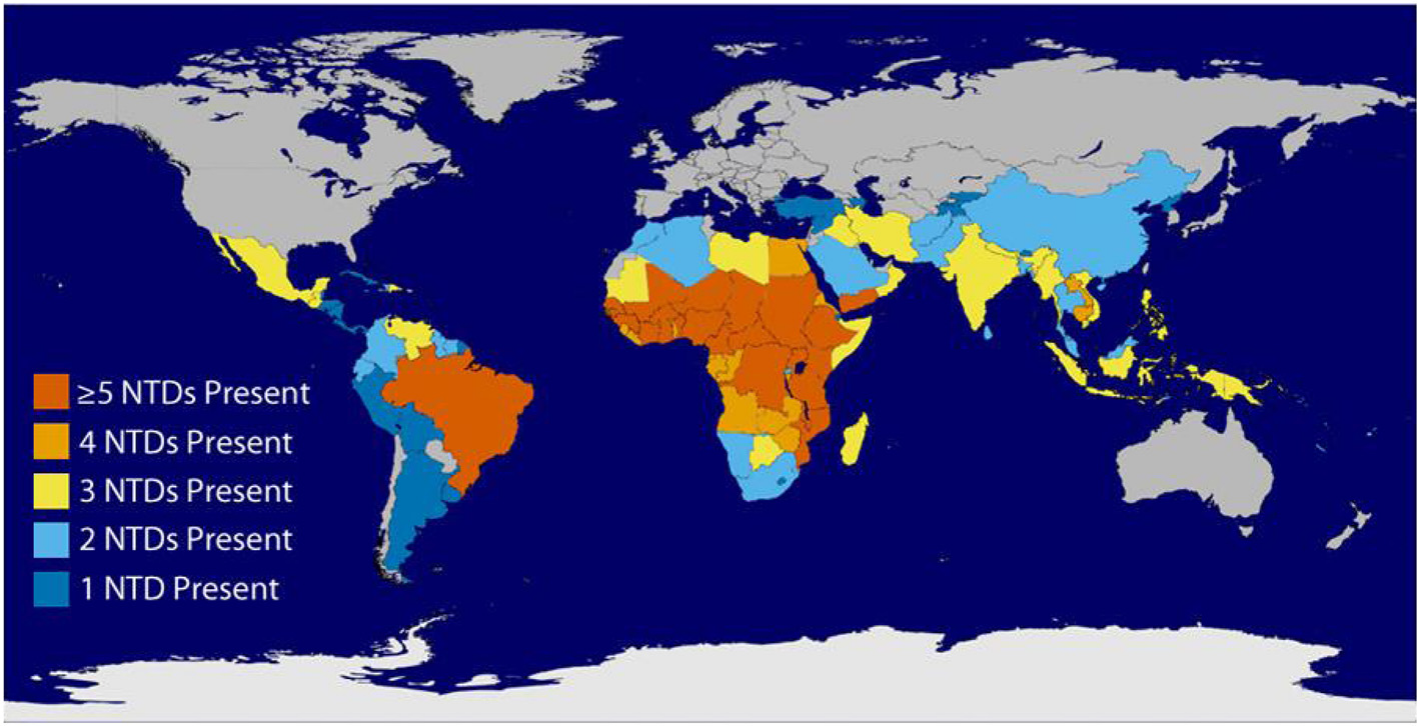
Global overlap of the six most common NTDs.

NTD are a group of 17 bacterial, parasitic protozoal, and viral infections (including dengue, rabies, chagas disease, human African trypanosomiasis, leishmaniasis, cysticercosis/taeniasis, dracunculiasis, echinococcosis, foodborne trematodiases, treponematoses, lymphatic filariasis, onchocerciasis, schistosomiasis, soil-transmitted helminthiases, buruli ulcer, leprosy, trachoma, and yaws) that are chronic and particularly endemic amongst the population in tropical and subtropical regions (Figure 1) [15]. The most common NTDs are a group of helminthic infections affecting one-third of the almost three billion people living on less than USD $2 per day in developing regions of Sub-Saharan Africa, Asia, and the Americas [8,16].

In 2012, malaria was responsible for over 1.1 million deaths globally [2] and was endemic in 104 countries with substantial geographic disparities. Around 81% of the malaria incidence and 91% of the malaria deaths in 2010 occurred in Africa and 86% involved children under five years of age [17]. A number of factors account for the malaria burden in developing countries, which include climate change, infrastructure, emerging drug and insecticide resistance, massive population and demographic shifts, and costs of containment and therapy. Malaria transmission also greatly depends on climatic conditions such as rainfall patterns, temperature, and humidity. In many endemic areas, transmission is seasonal, with peaks during and just after the rainy season. However, the past decade has seen tremendous expansion in malaria financing and consequent coverage of interventions. Approximately half of countries with ongoing malaria transmission are on track to meet the World Health Assembly (WHA) and Roll Back Malaria (RBM) targets to achieve a 75% reduction in malaria cases by 2015, as compared to those in 2000 [18].

More than 65 million people have been infected with HIV and 30 million people have died due to AIDS-related causes since the emergence of AIDS in 1981 [19]. In 2010, HIV was responsible for approximately 1.5 million deaths [2]. It has an extremely uneven geographical distribution, with Sub-Saharan Africa bearing more than two-thirds of the global burden [20], followed by Asia and the Pacific, where nearly 372,000 people became newly infected in 2011 [21]. Of the 34 million people living with HIV as of 2011, 3.3 million were children under 15 years and 16.7 million were women [21]. Adolescents are also vulnerable as an estimated 2.1 million adolescents (aged 10–19 years) were living with HIV in 2012 in LMICs, with the prevalence among young women twice as high as that among young men throughout Sub-Saharan Africa [22]. Progress has been made on some fronts. The *UNAIDS World AIDS Day Report 2012* reported a 50% reduction in HIV incidence in 25 LMICs between 2001 and 2011 [21], while in Sub-Saharan Africa, the number of newly infected children declined by 24% between 2009 and 2011 [20]. With this burden of HIV, the susceptibility of co-infection with leishmaniasis and TB also rises, and despite proper treatment, relapse is common and often results in death [23].

TB is the second greatest killer worldwide due to a single infectious agent after HIV/AIDS. In 2010, 1.2 million deaths were attributable to TB [2]. Over 95% of TB deaths occur in LMICs with the highest burden in Asia and Africa. The African region has 24% of the world’s TB cases and the highest rates of cases and deaths per capita, while India and China together account for almost 40% of the world’s TB burden [24]. There is also the emerging issue of multi-drug resistant TB (MDR-TB), which is rising and reached 60,000 in the 27 high MDR-TB burden countries worldwide in 2011 [24]. Although the MDG target to halt and reverse the TB epidemic by 2015 is already achieved, the disease burden remains enormous with resurgence in many areas due to HIV/AIDS. An estimated 13% of the TB cases in 2011 were co-infected with HIV and 430,000 deaths were among the HIV-positive population [24].

As a group, IDoP are amongst the top ten causes of disability-adjusted years (DALYs) (see Table 1). They can lead to burdensome health consequences that include blindness due to onchocerciasis and trachoma, and disfigurement from Lymphatic filariasis (LF), leishmaniasis, leprosy and buruli ulcer, leading to severe economic costs [25]. Schistosomiasis can result in severe organ pathology, anemia, malnutrition, and can also increase the risk of HIV. Repeated infection with trachoma can lead to scarring of the inside of the eyelid such that it turns inward, resulting in trichiasis and scar of the cornea. If untreated, irreversible corneal opacities are formed and blindness ensues. Ascariasis, trichuriasis, schistosomiasis, hookworms, malaria, and TB can lead to malnutrition and anemia, which is of particular concern for young children and pregnant women who are especially prone to the adverse health consequences of undernutrition. Most of these infections affect children and young adults leading to the loss of their most productive years, and some of these diseases can be fatal if left untreated [6]. Infections of severe intensity can impair physical growth and cognitive development and are a cause of micronutrient deficiencies leading to poor school performance and absenteeism in children, reduced work productivity in adults, and adverse pregnancy outcomes [26]. Malaria during pregnancy may lead to severe disease, spontaneous abortions, preterm birth, low birth weight, and anemia.

**Table 1:** Estimated number of disability-adjusted life years (DALYs) (in thousands) by IDoP

**Infectious diseases of poverty**	**Estimated DALYs (in thousands)**
***Neglected tropical diseases***	
Viral: Dengue	1, 243
Rabies	2, 297
Protozoan: Human African trypanosomiasis	1, 346
Chagas disease	499
Leishmaniasis	3, 754
Helminth: Cysticercosis/Taeniasis	503
Dracunculiasis	–
Echinococcosis	600
Food borne trematodiasis	665
Lymphatic filariasis	2, 740
Onchocerciasis	564
Schistosomiasis	3, 971
Ascariasis	1, 254
Trichuriasis	630
Hookworm disease	3, 159
Bacterial: Buruli ulcer	–
Leprosy	215
Trachoma	308
Yaws	–
***Tuberculosis***	42,240
***HIV/AIDS***	95,226
***Malaria***	55,414

*Source*: WHO. Disease Burden: DALY Estimates 2011 and Global Burden of Diseases 2010.

In addition to the grave health consequences, this group of diseases also leads to huge economic costs for both the individual and society. Leishmaniasis results in approximately USD $1.3 billion/years lost in productivity, while for trachoma the loss in productivity is estimated at USD $2.9 billion [27]. In India, the average total economic burden for dengue fever is approximately USD $29.3 million, and schistosomiasis in the Philippines results in 45.4 days off-work lost per infected person/year [23]. Direct costs associated with malaria-encompassing illnesses, treatment, and premature deaths have been estimated to be at least USD $12 billion per year, however, the costs are often more than that in terms of lost economic growth [27]. Conversely, controlling these diseases has the potential to increase productivity as deworming against soil-transmitted helminthiasis (STH) in Kenya has shown to increase the present wages by more than USD $40 per treated person, with a benefit-to-cost ratio of 100 [28].

### Interventions and coverage

The World Health Organization (WHO) promotes the use of five public health strategies to control, eliminate, and eradicate NTDs. These include preventive chemotherapy; innovative and intensified disease-management; vector control and pesticide management; provision of safe drinking water, basic sanitation and hygiene, and education; and veterinary public health services [29]. Mass drug administration (MDA) has been a major approach to combat helminthiasis including ascariasis, trichuriasis, hookworm, schistosomiasis, LF, onchocerciasis, and trachoma in developing countries [30]. There is evidence suggesting significant benefits of treating diagnosed cases [31,32], and these medicines are not only effective but also cost efficient. A USD $0.50 package containing several inexpensive and safe drugs can treat more than 15 types of neglected parasitic infections [33].

The WHO recommends periodic preventive treatment with anthelmintics for all at-risk people living in endemic areas to reduce morbidity by reducing the worm burden. Large-scale and successful control activities implemented during 2001–2010 demonstrate the feasibility of mass deworming, and these experiences have informed the development of tools to facilitate its implementation [34]. Of the 123 countries requiring preventive chemotherapy, 40 require interventions for three or more diseases and 33 of these 40 countries are in Africa [23,29]. About 1.9 billion people require preventive chemotherapy for at least one NTD; 55% of them require preventive treatment for one or two diseases, and 45% require it for three or more diseases [23,29]. However, by the end of 2010, only 25 countries had achieved at least one of the targets set for delivering preventive chemotherapy for LF, onchocerciasis or STH, and only five of these countries had reached the targets for delivering preventive chemotherapy for three or more diseases simultaneously [23,29]. Therefore, interventions need to be scaled-up considerably if targets set in the WHA’s resolutions are to be met [29].

Key interventions recommended by the WHO to prevent and control malaria include prompt and effective treatment with artemisinin-based combination therapies; the use of insecticide-treated nets (ITNs); and indoor residual spraying (IRS) with insecticide to control vector mosquitoes. In the past decade, the percentage of households owning at least one ITN in Sub-Saharan Africa reached an estimated 53% by 2011 and remained at 53% in 2012. It must be noted however that this is greatly challenged by the limited deliveries of ITNs and increasing mosquito resistance to insecticides [18]. In 2011, the proportion of pregnant women attending an antenatal care clinic and receiving two doses of intermittent preventive treatment during pregnancy (IPTp) ranged from 30% to 57% in 2011, however, intermittent preventive treatment for infants and seasonal malaria chemoprevention for children still await to be adopted by endemic countries [18,35]. An emerging challenge arising due to over diagnosis and treatment is the drug resistance to artemisinins detected in four countries of the Greater Mekong Subregion: Cambodia, Myanmar, Thailand, and Vietnam [18]. More recently, the WHO has introduced a new initiative, entitled “T3: Test, Treat and Track”. It urges malaria-endemic countries and donors to move towards universal access to diagnostic testing and antimalarial treatment to build robust control and surveillance systems. The current recommendation of diagnostic confirmation of malaria before treatment initiation was adopted by 41 countries in the African region, however, this practice attained less than 50% coverage in 2011 even in public health facilities [35].

Strategies for HIV prevention involve risk-reduction through education and counseling. The WHO has recommended key approaches which include condom use, testing and counseling, male circumcision, preventive antiretroviral therapy (ART), harm reduction for injecting drug users, and elimination of mother-to-child transmission (MTCT) of HIV [36,37]. In 2012, 9.7 million people in LMICs received ART (representing 61% of all who were eligible), however, under the 2013 WHO guidelines, this treatment coverage represents only 34% of the 28.3 million people eligible [22]. Although the coverage of effective ART regimens in LMICs for preventing MTCT was 57% in 2011, a lot is still desired to eliminate it completely as a recent report suggested that, on average, nearly half of all children newly infected with HIV in the 20 African countries surveyed were acquiring HIV during breastfeeding because of low ART coverage during this period. In 2012, 375,000 more pregnant women living with HIV received ART medicines than in 2009 [38].

TB is preventable as well as curable, and its transmission could be prevented by prompt identification and treatment of the infection. The WHO is working to dramatically reduce the burden of TB and halve TB deaths and prevalence by 2015 through its Stop TB Strategy and by supporting the Global Plan to Stop TB. Between 1995 and 2011, 51 million people were successfully treated for TB in countries that had adopted the WHO strategy, saving 20 million lives [24]. There has also been progress in implementing collaborative TB/HIV activities as recommended by the WHO in 2004 [24].

### The changing care paradigm

A large proportion of infectious diseases in LMICs are entirely avoidable or treatable with existing medicines or interventions which are also highly cost effective, however, their delivery to the affected populations has proven very difficult due to weak health systems and infrastructures [14]. Another major issue is access to and utilization of health services, which has been a concern in LMICs, with not enough progress being made on various health parameters. Other impeding factors include gender discrimination, low levels of female literacy, and lack of women empowerment; all of these prevent women from seeking care for themselves and their children. Health care is also unaffordable for many families due to economic barriers including formal and informal healthcare fees, cost of medicines and tests, cost of not working during hospitalization, travel, food, and accommodation.

Effective delivery of proven interventions require a variety of components ranging from training health workers, effective use of epidemiological data, proper delivery of safe medicines and commodities, accurate monitoring and evaluation, and providing feedback to the community. Successful implementation requires a positive interrelation between programs for disease control and the health system at large. Global health initiatives have created a complex health system with an increasing number of actors entering the field and implementing diverse health systems strategies [39]. These health system issues need to be acknowledged and worked on [40]. Therefore, a combination of public health strategies is required to achieve control of each of these diseases and an integrated approach to disease control and elimination is strongly advised especially in high-burden countries [29]. There are also wide inequities in access to and utilization of healthcare amongst the wealth quintiles. Poor children are more likely to be exposed to health risks, and they have less resistance to diseases because of undernutrition and other hazards. These inequities are compounded by reduced access to preventive and curative interventions [41].

Community-based interventions (CBIs) have the potential to overcome the barriers of access and availability and, if adequately equipped and supported by parallel structures, can make a significant impact on reducing the burden of IDoP [42-44]. However, a major issue is the availability of a trained health force to scale-up these interventions in population settings. According to a 2006 report by the WHO, 57 countries from Africa and Asia are facing shortages of a healthcare workforce and a total estimate of 4,250,000 workers are needed to fill the gap [45]. For scaling-up these interventions, there is a need to strengthen health systems [46], and also to develop alternative cadres for task shifting from trained healthcare workers to lay workers and from specialized facilities to community-based delivery [47]. Many of the interventions targeting infectious diseases have been administered via community platforms through the community health workers (CHWs) who have received basic training [48-50]. Although CHWs may not be able to replace the need for sophisticated healthcare delivery, they can play an important role in providing access to health care and services for the unreachable. Successful examples exist, for example, in Brazil, where CHWs provide coverage to over 60 million people [51]. Ethiopia is training about 30,000 workers with emphasis on maternal and child health, HIV, and malaria. Other similar programs are also being considered in countries such as India, Ghana, and South Africa. Apart from providing chemotherapy, CHWs can also play a major role in imparting health education regarding general hygiene and sanitation and intervene for vector control measures within household and community settings. These community delivery strategies are not only effective but are also cost efficient, and by training teachers and other school personnel to administer anthelmintic drugs, costs could be reduced by “piggy-backing” on existing programs in the educational sector [52]. In Ghana and Tanzania, delivery of school-based targeted anthelmintic treatment cost as little as US $0.03 per child, which is as low as one-tenth of the estimated costs for vertical delivery [52].

## Conclusion

In order to evaluate the effectiveness of CBIs, we developed an analytical framework and conducted systematic reviews of the existing studies focusing on CBIs for prevention and control of helminthic and non-helminthic NTDs, malaria, TB, and HIV/AIDS compared to the routine healthcare delivery. For this review, we categorized NTDs into helminthic and non-helminthic diseases, and reported the findings accordingly in separate papers. Helminthic diseases included soil-transmitted helminthiasis (ascariasis, hookworm, and trichuriasis) along with schistosomiasis, lymphatic filariasis, onchocerciasis, and dracunculiasis. Non-helminthic diseases included dengue, African trypanosomiasis, chagas, leishmaniasis, trachoma, leprosy, and buruli ulcer. In this series of eight papers, we describe the analytical framework and methodology used for the systematic reviews, and report findings on the effectiveness of CBIs for the prevention and control of helminthic NTDs, non-helminthic NTDs, malaria, HIV/AIDS, and tuberculosis. In the final paper, we propose a way forward.

## Abbreviations

ART: Antiretroviral therapy; CBI: Community-based intervention; CHW: Community health worker; DALY: Disability-adjusted life year; HIV/AIDS: Human immunodeficiency virus/acquired immunodeficiency syndrome; IDoP: Infectious disease of poverty; IPTp: Intermittent preventive therapy during pregnancy; IRS: Indoor residual spraying; ITN: Insecticide-treated net; LF: Lymphatic filariasis; LMIC: Low- middle- income country; MDA: Mass drug administration; MDG: Millennium development goal; MDR-TB: Multi drug resistant tuberculosis; MTCT: Mother-to-child transmission; NTD: Neglected tropical disease; RBM: Roll back malaria; STH: Soil-transmitted helminthiasis; TB: Tuberculosis; UN: United nations; WHA: World health assembly; WHO: World health organization.

## Competing interests

The authors declare that they have no financial or non-financial competing interests.

## Authors’ contributions

ZAB and JS were responsible for designing and coordinating the review. All authors contributed, read and approved the final manuscript.

## Supplementary Material

Additional file 1 Multilingual abstracts in the six official working languages of the United Nations. Click here for file
