# Supplementary material for: Global burden, distribution, and interventions for infectious diseases of poverty
Source: Infect Dis Poverty. 2014 Jul 31;3:21. doi: 10.1186/2049-9957-3-21 (PMC4126350; doi:10.1186/2049-9957-3-21)
Supplement: Additional file 1 — Multilingual abstracts in the six official working languages of the United Nations. [file 2049-9957-3-21-S1.pdf]

Translation of the abstract into the six official working languages of the United Nations

العبء، والتوزيع والتدخلات العالمية لمكافحة أمراض الفقر المعدية

ذو الفقار أ بوتا، جوهانس سومرفلد، زهره س لاسي، ريحانه أ سلام، جاي ك داس

تلخيص

تؤثر أمراض الفقر المعدية (IDoPs) بشكل غير متناسب على السكان الأكثر فقراً في العالم وتسهم في دورة الفقر نتيجة لانخفاض الإنتاجية الناجمة عن طول فترة المرض، والعجز والوصمة الاجتماعية. على الصعيد العالمي، في عام 2010، زادت الوفيات الناجمة عن فيروس نقص المناعة البشرية (HIV)/ متلازمة نقص المناعة المكتسب (AIDS) إلى 1.5 مليون؛ ارتفعت وفيات الملاريا إلى 1.17 مليون، وارتفعت وفيات الأمراض المدارية المهملة إلى 152,000 بينما قتل السل (TB) 1.2 مليون شخص في 2010. تكمن الاختلافات الإقليمية الكبيرة في توزيع هذه الأمراض لأنها تتركز أساساً في المناطق الريفية في أفريقيا جنوب الصحراء الكبرى، وآسيا وأمريكا اللاتينية، مع تداخل جغرافي ومستويات عالية من الإصابة المشتركة. هناك دليل على تدخلات للوقاية ومكافحة هذه الأمراض؛ لكن التغطية مازالت منخفضة مع التحدي الناتج من مقاومة مضادات الميكروبات. لذلك، البرامج المقدمة والمتوفرة بالمجتمعات تكتسب الدعم بشكل متزايد لتضمن الاستدامة ومكافحة الإصابات المشتركة.

بسبب عبء ارتفاع معدلات الإصابة بالمرض والوفيات لهذه الأمراض، خصوصاً في البيئات الفقيرة الموارد، لابد من إجراء مراجعة منهجية لتحديد استراتيجيات للوقاية ومكافحة هذه الأمراض. لذلك، قد حاولنا تقييم فعالية واحدة من هذه الاستراتيجيات التي تطبق بالمجتمعات وأثرها على الوقاية وعلاج أمراض الفقر المعدية. في هذا البحث نقوم بتوصيف العبء، وعلم الأوبئة والتدخلات المحتملة لأمراض الفقر المعدية. في حين نقوم في الأبحاث اللاحقة من هذه السلسلة بتوصيف الإطار التحليلي والمنهج المتبع في توجيه المراجعات المنهجية، وتقديم تقرير عن النتائج وشرح تحليلنا لتأثير الاستراتيجيات الموجودة بالمجتمعات على الإصابات الفردية بأحد أمراض الفقر المعدية.

Translated from English version into Arabic by Saher Salama, through

TRANSLATORS
WITHOUT BORDERS

贫困所致传染病的全球疾病负担、分布和干预

Zulfiqar A Bhutta, Johannes Sommerfeld, Zohra S Lassi, Rehana A Salam, Jai K Das

摘要

贫困所致传染病不均衡地侵袭世界最贫困的人群并造成世代贫穷（贫困恶性循环），从而造成生产力降低并伴随长期患病、残疾和社会耻辱。2010 年，全球因人体免疫缺损病毒(HIV)/获得性免疫缺陷综合症(AIDS)致死人数上升至 1 500 000, 疟疾致死人数上升至 1 170 000, 被忽视热带病致死人数上升至 152 000，其中结核 2010 年致 1 200 000 人死亡。这些疾病的分布存在巨大的地区差异，主要集中在撒哈拉以南非洲、亚洲和拉丁美洲的农村地区,，并伴随地理位置重叠和高感染度的合并感染。

询证健康干预用于防控这些疾病，但覆盖率仍低，并面临抗药性的新挑战。因此，愈加提倡利用以社区为基础的交付平台，以确保可持续性和对抗合并感染。

由于这些疾病的高发病率和死亡率负担，特别是在资源匮乏的环境中，有必要进行系统综述以论证这些疾病预防和控制策略。因此，我们试图对社区干预策略进行有效性评估，并评价其对预防和治疗贫困所致传染病的影响。在本文中，我们描述了贫困所致传染病的疾病负担、流行病学和潜在的干预措施。在随后的本系列论文中，我们描述所使用的分析框架和方法学，该框架和方法学用于指导系统综述，并报告我们通过社区干预措施对具体的贫困所致传染病的影响所分析得出的发现和解释。

Translated from English version into Chinese by Zheng Qi, through

CHINESE CENTER FOR DISEASE CONTROL AND PREVENTION

NATIONAL INSTITUTE FOR PARASITIC DISEASES

Poids mondial des maladies infectieuses liées à la pauvreté, distribution et interventions

Zulfiqar A Bhutta, Johannes Sommerfeld, Zohra S Lassi, Rehana A Salam, Jai K Das

Résumé

Les maladies infectieuses liées à la pauvreté (MILP) affectent en priorité les populations les plus pauvres du monde, contribuant ainsi, par la perte de productivité résultant d'un mauvais état de santé prolongé, du handicap et de la stigmatisation sociale, à l'entretien du cycle de la pauvreté. Sur l'année 2010, le nombre de morts causées par le virus de l'immunodéficience humaine (VIH) et le syndrome d'immunodéficience acquise (SIDA) a atteint 1,5 million, la mortalité due au paludisme 1,17 million de morts, les maladies tropicales négligées ont fait 152 000 mots et la tuberculose 1,2 million. Il existe d'importantes variations régionales dans la distribution de ces maladies, qui se concentrent principalement dans les zones rurales d'Afrique subsaharienne, d'Afrique et d'Amérique Latine, où leurs territoires se recoupent avec pour conséquence des taux élevés de co-infections. Des interventions basées sur les preuves permettent de prévenir ces maladies et de les combattre, mais elles restent peu étendues et elles se heurtent à l'émergence de résistances aux antimicrobiens. Les plates-formes de prestations communautaires sont donc de plus en plus souvent préconisées pour assurer la durabilité des interventions et lutter contre les co-infections.

En raison de la forte morbidité et mortalité liée à ces maladies, en particulier dans les zones à faibles ressources, une revue systématique apparaît impérativement nécessaire pour identifier les stratégies qui permettront de les prévenir et de les combattre. Nous avons donc tenté d'évaluer l'efficacité de l'une de ces stratégies d'intervention communautaire et son impact sur la prévention et le traitement des MILP. Nous décrivons ici le poids et l'épidémiologie des MILP ainsi que les interventions envisageables pour les combattre. Dans les articles suivants de cette série, nous décrivons le cadre analytique et la méthodologie employée pour guider les revues systématiques et rendons compte des résultats et des interprétations de notre analyse de l'impact des stratégies communautaires sur certaines MILP.

Translated from English version into French by Suzanne Assenat, through

TRANSLATORS
WITHOUT BORDERS

Глобальное бремя, распространение и профилактика инфекционных болезней бедности

Зульфикар А. Бхутта, Йоханнес Зоммерфельд, Зохра С. Ласси, Рехана А. Салам, Джай К. Дас

Аннотация

Инфекционные болезни бедности (ИББ) диспропорционально поражают беднейшие слои мирового населения и вносят свой вклад в создание порочного круга бедности в результате снижения работоспособности из-за долгой болезни, инвалидности и социальной стигматизации. В мировом масштабе в 2010 г. количество случаев смерти от вируса иммунодефицита человека (ВИЧ)/синдрома приобретенного иммунодефицита (СПИД) выросло до 1,5 млн.; смертность от малярии увеличилась до 1,17 млн., смертность от ЗТБ — до 152000, а от туберкулеза (ТБ) в 2010 году погибло 1,2 миллиона человек. В распространении этих болезней существуют значительные региональные различия, поскольку они обычно сосредоточены в сельских районах Тропической Африки, Азии и Латинской Америки, с географическим перекрыванием и высоким уровнем множественного инфицирования. Существуют способы профилактики, сформировавшиеся на основе опыта, которые способны предотвращать и контролировать распространение этих болезней; тем не менее, охват населения остается небольшим, а резистентность антимикробным препаратам начинает представлять собой серьезную проблему. Поэтому все большее влияние уделяется программам на основе общин, которые обеспечивают устойчивую работу программы и эффективно борются со множественными инфекциями.

Из-за большого количества осложнений и смертности при данных заболеваниях, особенно в бедных районах, чрезвычайно важно вести систематические наблюдения, чтобы разработать стратегии по профилактике и контролю этих заболеваний. Поэтому мы попытались оценить эффективность одной из этих стратегий, действующих на основе общины, и определить ее влияние на профилактику и лечение ИББ. В данной работе мы описываем глобальное бремя, эпидемиологию и возможные способы профилактики ИББ. В последующих работах этой серии мы описываем аналитические рамки и методологию, использованную при периодических обзорах, а также сообщаем о находках и

своей интерпретации нашего анализа влияния стратегий, действующих на основе общины, на отдельные ИББ.

Translated from English version into Russian by Mayya Shlyakhter, through

TRANSLATORS
WITHOUT BORDERS

Carga, distribución e intervenciones globales para las enfermedades infecciosas de la pobreza

Zulfiqar A Bhutta, Johannes Sommerfeld, Zohra S Lassi, Rehana A Salam, Jai K Das

Sinopsis

Las enfermedades infecciosas de la pobreza (IDoPs, por sus siglas en inglés) afectan de una manera desproporcionada a las poblaciones más pobres del mundo y contribuyen a un ciclo de pobreza como resultado de la disminución de la productividad resultante de enfermedades a largo plazo, la discapacidad y el estigma social. A nivel mundial, en el año 2010, las muertes por virus de inmunodeficiencia humana (VIH) / Síndrome de Inmunodeficiencia Adquirida (SIDA) aumentaron a 1 • 5 millones de dólares; la mortalidad por malaria se elevó a 1 • 17 millones, la mortalidad por EID se elevó a los 152.000, mientras que la tuberculosis (TB) mató a 1.2 millones de personas. Existen variaciones regionales sustanciales en la distribución de estas enfermedades, ya que se concentran principalmente en las zonas rurales del África subsahariana, Asia y América Latina, con superposición geográfica y altos niveles de coinfección. Existen intervenciones basadas en la evidencia para prevenir y controlar estas enfermedades; sin embargo, la cobertura sigue siendo baja con un nuevo desafío de la resistencia antimicrobiana. Por lo tanto, las plataformas de entrega basadas en la comunidad están siendo propuestas cada vez más para garantizar la sostenibilidad y combatir las coinfecciones.

Debido a la alta carga de morbilidad y mortalidad de estas enfermedades, especialmente en entornos con escasos recursos, es imprescindible llevar a cabo una revisión sistemática para identificar las estrategias de prevención y controlar estas enfermedades. Nosotros, por lo tanto, hemos tratado de evaluar la eficacia de una de estas estrategias, que es la entrega basada en la comunidad y su impacto en la prevención y el tratamiento de las IDoPs. En este trabajo se describe la carga, la epidemiología y las posibles intervenciones para las IDoPs. Mientras que en los artículos posteriores de la serie se describen el marco analítico y la metodología utilizada para guiar las revisiones sistemáticas e informar de los resultados y de la interpretación de nuestros análisis del impacto de las estrategias basadas en la comunidad en una IDoP particular.

Translated from English version into Spanish by Eva Castilla, through

TRANSLATORS
WITHOUT BORDERS
